# Age – related treatment strategy and long-term outcome in acute myocardial infarction patients in the PCI era

**DOI:** 10.1186/1471-2261-12-31

**Published:** 2012-04-25

**Authors:** Petr Kala, Jan Kanovsky, Richard Rokyta, Michal Smid, Jan Pospisil, Jiri Knot, Filip Rohac, Martin Poloczek, Tomas Ondrus, Maria Holicka, Jindrich Spinar, Jiri Jarkovsky, Ladislav Dusek

**Affiliations:** 1 Department of Internal Medicine and Cardiology, University Hospital Brno and Faculty of Medicine, Masaryk University, Brno, Czech Republic; 2 Department of Cardiology, University Hospital Plzen and Faculty of Medicine Plzen, Charles University Prague, Prague, Czech Republic; 3 Cardiocenter, 3rd Faculty of Medicine, Charles University Prague, Prague, Czech Republic; 4 Institute of Biostatistics and Analyses, Faculty of Science and Faculty of Medicine, Masaryk University, Brno, Czech Republic

## Abstract

**Background:**

Older age, as a factor we cannot affect, is consistently one of the main negative prognostic values in patients with acute myocardial infarction. One of the most powerful factors that improves outcomes in patients with acute coronary syndromes is the revascularization preferably performed by percutaneous coronary intervention. No data is currently available for the role of age in large groups of consecutive patients with PCI as the nearly sole method of revascularization in AMI patients. The aim of this study was to analyze age-related differences in treatment strategies, results of PCI procedures and both in-hospital and long-term outcomes of consecutive patients with acute myocardial infarction.

**Methods:**

Retrospective multicenter analysis of 3814 consecutive acute myocardial infarction patients divided into two groups according to age (1800 patients ≤ 65 years and 2014 patients > 65 years). Significantly more older patients had a history of diabetes mellitus and previous myocardial infarctions.

**Results:**

The older population had a significantly lower rate of coronary angiographies (1726; 95.9% vs. 1860; 92.4%, p < 0.0001), PCI (1541; 85.6% vs. 1505; 74.7%, p < 0.001), achievement of optimal final TIMI flow 3 (1434; 79.7% vs. 1343; 66.7%, p < 0.001) and higher rate of unsuccessful reperfusion with final TIMI flow 0-1 (46; 2.6% vs. 78; 3.9%, p = 0.022). A total of 217 patients (5.7%) died during hospitalization, significantly more often in the older population (46; 2.6% vs. 171; 8.5%, p < 0.001). The long-term mortality (data for 2847 patients from 2 centers) was higher in the older population as well (5 years survival: 86.1% vs. 59.8%). Though not significantly different and in contrast with PCI, the presence of diabetes mellitus, previous MI, final TIMI flow and LAD, as the infarct-related artery, had relatively lower impact on the older patients. Severe heart failure on admission (Killip III-IV) was associated with the worst prognosis in the whole group of patients, though its significance was higher in the youngers (HR 6.04 vs. 3.14, p = 0.051 for Killip III and 12.24 vs. 5.65, p = 0.030 for Killip IV). We clearly demonstrated age as a strong discriminator for the whole population of AMI patients.

**Conclusions:**

In a consecutive AMI population, the older group (>65 years) was associated with a less pronounced impact of risk factors on long-term outcome. To ascertain the coronary anatomy by coronary angiography and proceed to PCI if suitable regardless of age is crucial in all patients, though the primary success rate of PCI in the older age is lower. Age, when viewed as a risk factor, was a dominant discriminating factor in all patients.

## Background

Over the past decades the incidence of acute myocardial infarction (AMI) together with mortality have decreased dramatically in developed countries [1,2]. These favorable trends reflect an improvement in many factors that influence outcomes in patients with acute coronary syndromes (ACS) [3]. Older age, as a factor we cannot affect, is consistently one of the main negative prognostic values in most trials [4,5]. One of the most powerful factors that improves outcomes in patients with ACS is the revascularization preferably performed by percutaneous coronary intervention (PCI) [6,7]. No data is currently available for the role of age in large groups of consecutive patients with PCI as the nearly sole method of revascularization in AMI patients. The aim of this study was to assess age related differences in treatment strategies (conservative or invasive), results of PCI procedures and both in-hospital and long-term outcomes in AMI patients.

## Methods

### Patients’ group and data collection

This multicenter, retrospective project included 3814 consecutive “all-comer” patients with a diagnosis of AMI. Age under 18 was the only exclusion criterion. Patients were enrolled in 3 tertiary complex cardiovascular university centers providing the 24/7 catheterization service (3 year period, 2005 to 2007 in two centers, and a two year period 2007 to 2008 in one center). All patients with a final diagnosis of acute myocardial infarction with/without ST elevations (STEMI / NonSTEMI) were included in the registry. The diagnosis of AMI was based on the ESC/ACC/AHA definition [8] and had to be confirmed at the time of discharge from the hospital, or post-mortem, if a patient died during hospitalization.

Admission and discharge reports of all patients were analyzed and transferred to a registry created for the project. Following parameters were collected: 1) History of diabetes and previous MI; 2) Clinical data, particularly Killip class on admission; 3) 12-lead ECG regarding the presence of ST segment changes and bundle branch blockades at the time of admission (Table 1); 4) Coronary angiography including the number of diseased vessels, initial and final Thrombolysis In Myocardial Infarction (TIMI) flow and determination of the infarct-related artery (IRA) (left anterior descending artery = LAD, left circumflex artery = LCX and right coronary artery = RCA or the disease of the left main coronary artery (LMCA) described separately) (Table 1); 5) Left ventricular ejection fraction (LVEF), assessed through echocardiography before hospital discharge or alternatively through LV angiography during catheterization. No thrombolytic therapy was used during the assessed period of time. The success of PCI was defined as a complete reperfusion of IRA represented by a final TIMI score of 3.

**Table 1:** Age-related baseline differences

**Age**	**≤65 years**	**>65 years**	**p**
Number of patients	1800	2014	NA
**Patients’ characteristics**			
Males	1454 (80.8%)	1132 (56.2%)	<0.001
History of DM	389 (21.6%)	763 (37.9%)	<0.001
History of previous MI	268 (14.9%)	505 (25.1%)	<0.001
**Killip class on admission**			
I	1501 (83.4%)	1361 (67.6%)	<0.001
II	179 (9.9%)	410 (20.4%)	<0.001
III	54 (3.0%)	131 (6.5%)	<0.001
IV	66 (3.7%)	112 (5.6%)	0.006
**Admission ECG**			
STEMI + new LBBB	1060 (58.9%)	956 (47.5%)	<0.001
NonSTEMI	740 (41.1%)	1058 (52.5%)	<0.001
**CAG**			
CAG	1726 (95.9%)	1860 (92.4%)	<0.001
No indication for CAG	74 (4.1%)	154 (7.6%)	<0.001
**Number of diseased vessels**			
Single vessel disease	717 (39.8%)	463 (23.0%)	<0.001
Two vessel disease	506 (28.1%)	546 (27.1%)	0.491
Three vessel disease	478 (26.6%)	807 (40.1%)	<0.001
Left main artery disease	25 (1.4%)	44 (2.2%)	0.069
**IRA (by CAG or autopsy)**			
Left main	26 (1.4%)	42 (2.1%)	0.143
LAD	660 (36.7%)	763 (37.9%)	0.441
LCX	360 (20.0%)	293 (14.5%)	<0.001
RCA	585 (32.5%)	562 (27.9%)	0.002
ACB	14 (0.8%)	25 (1.2%)	0.197
Not known	155 (8.6%)	329 (16.3%)	<0.001
**Initial TIMI flow**			
TIMI 0-1	877 (48.7%)	781 (38.8%)	<0.001
TIMI 2	288 (16.0%)	315 (15.6%)	0.790
TIMI 3	480 (26.7%)	589 (29.2%)	0.077
**PCI**			
PCI total number	1541 (85.6%)	1505 (74.7%)	<0.001
No PCI	259 (14.4%)	509 (25.3%)	<0.001
**PCI% of CAG**	1541/1726 (89.3%)	1505/1860 (80.9%)	<0.001

Numbers in the second and third column represent absolute numbers of patients and percentage of the related group. Statistically significant difference is p < 0.05.

*DM* diabetes mellitus, *MI* myocardial infarction, *ECG* electrocardiogram, *STEMI* ST segment elevation myocardial infarction, *LBBB* left bundle branch block, *NonSTEMI* non-ST elevation myocardial infarction.

The World Heart Organization (WHO) definition for age reflecting also the most common retirement age in Europe was used for creating two groups of patients. The first group (younger population) included subjects ≤ 65 years of age, the second group (older population) included subjects > 65 years of age. Endpoints for this analysis were as follows: coronary angiography and PCI performed during index hospitalization, final TIMI flow after PCI, a change in TIMI flow during the PCI procedure and in-hospital mortality. Long-term mortality data independently followed by the Czech Ministry of Health were available from 2 centers. All data in the registry were anonymised and the study was provided in compliance with the Helsinki Declaration. According to the national law no ethics committee approval or signed patient informed consent were needed.

### Statistical analysis

Categorical parameters were described by absolute and relative frequency of categories. Continuous parameters were described using the median and the 5^th^ – 95^th^ percentile range. Statistical significance of differences between groups of patients was analyzed using the Mann-Whitney *U* test for continuous variables and the Fisher exact test for categorical variables. Risk factors associated with long-term survival were evaluated using the Cox proportional hazard model and described using hazard ratios and their 95% confidence interval. Survival data were visualized using the Kaplan-Meier methodology. Influence of patient age on HR values within the age categories was analyzed using the interaction term in the Cox proportional hazard model. P values < 0.05 were considered statistically significant.

## Results

The first group (1800 patients) included subjects ≤ 65 years of age, the second group (2014 patients) included subjects > 65 years of age. In the younger group there was a higher distribution of men, and a lower rate of diabetes mellitus and previous myocardial infarctions. Less younger patients presented with acute heart failure on admission to hospital. Consequently, Killip class II and III were more common in the older patients as well as cardiogenic shock, described as Killip class IV. STEMI was diagnosed more often in the younger group (Table 2).

**Table 2:** Age-related endpoints of the project

**Age**	**≤65 years**	**>65 years**	**p**
Number of patients	1800	2014	NA
**Final TIMI flow**			
TIMI 0-1	46 (2.6%)	78 (3.9%)	0.022
TIMI 2	64 (3.6%)	102 (5.1%)	0.026
TIMI 3	1434 (79.7%)	1343 (66.7%)	<0.001
1..3	118 (6.6%)	105 (5.2%)	0.084
0..3	655 (36.4%)	514 (25.5%)	<0.001
**In-hospital mortality**	**46 (2.6%)**	**171 (8.5%)**	**<0.001**

Numbers in the second and third column represent absolute numbers of patients and percentage of the related group, unless specified differently. Statistically significant difference is p < 0.05.

*CAG* Coronary Angiography, *PCI* Percutaneous Coronary Intervention, *TIMI* Thrombolysis In Myocardial Infarction.

**Figure 1:**
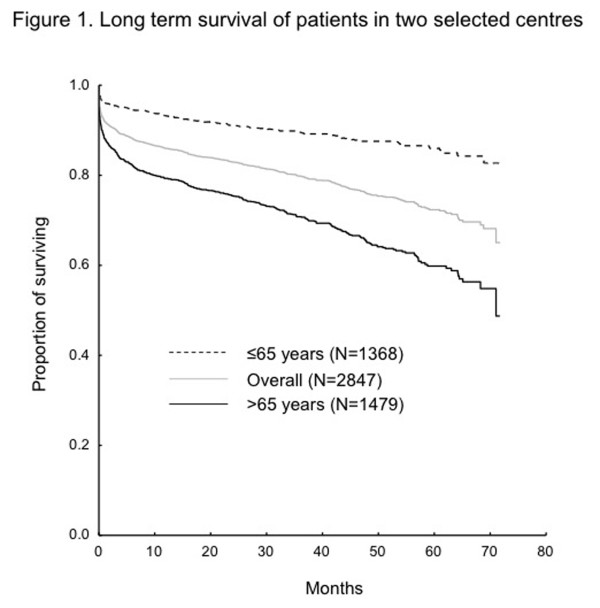
Long-term survival of patients in two selected centers.

More invasive therapeutic approach was observed in the younger patients in term of higher number of coronary angiographies (CAG) and PCI. In the older population there was a higher rate of unsuccessful reperfusions represented by a final TIMI score 0-1. A total of 217 patients (5.7%) died during hospitalization, significantly more in the older group (46; 2.6% vs. 171; 8.5%, p < 0.001) (Table 2). Long-term mortality (calculated from data for 2847 patients from 2 centers) was higher in the older patients as well (3 and 5 years survival supplemented by 95% confidence interval: 89.6% (87.9%; 91.2%) vs. 70.8% (68.4%; 73.2%) and 86.1% (83.7%; 88.4%) vs. 59.8% (56.4%; 63.2%) respectively, p < 0.001; log rank test) (Figure 1). Similar risk factors significantly influenced long term survival in both the younger and older population with only a limited number of differences (Table 3). Emergent coronary-artery bypass surgery (CABG) during the first 24 hours after admission was performed in 3.4% in total (2.0% in STEMI and 4.4% in NSTEMI). Significantly more older patients were treated using emergent CABG (4.1% vs. 2.5%, p = 0.019 ).

**Table 3:** Risk factors influencing long term survival of patients in two selected centers

							**Interaction**
	**N**	**HR (95% CI)**	**p**	**N**	**HR (95% CI)**	**p**	**p**
Age (10 years)	-	1.76 (1.35; 2.28)	<0.001	-	2.03 (1.75; 2.35)	<0.001	0.458
Men	1116	1.18 (0.78; 1.79)	0.441	856	0.90 (0.76; 1.08)	0.262	0.227
DM	303	1.91 (1.38; 2.65)	<0.001	574	1.41 (1.18; 1.68)	<0.001	0.085
Previous MI	219	2.02 (1.42; 2.87)	<0.001	385	1.46 (1.20; 1.76)	<0.001	0.189
Killip I	1114	*Basal category*		958	*Basal category*		
Killip II	155	2.52 (1.67; 3.81)	<0.001	334	1.80 (1.47; 2.22)	<0.001	0.298
Killip III	39	6.04 (3.48; 10.48)	<0.001	104	3.14 (2.36; 4.18)	<0.001	0.051
Killip IV	53	12.24 (7.94; 18.89)	<0.001	75	5.65 (4.14; 7.70)	<0.001	0.030
PCI	1169	0.60 (0.42; 0.88)	0.008	1109	0.46 (0.38; 0.55)	<0.001	0.202
No PCI	199	1.66 (1.14; 2.41)		370	2.19 (1.83; 2.63)		
STEMI + new onset of LBBB	948	1.02 (0.72; 1.43)	0.924	990	1.21 (1.00; 1.47)	0.056	0.312
NonSTEMI	420	0.98 (0.70; 1.38)		489	0.83 (0.68; 1.01)		
Final TIMI flow 2-3	1162	0.20 (0.10; 0.41)	<0.001	1096	0.32 (0.22; 0.47)	<0.001	0.151
Final TIMI flow 0-1	19	5.00 (2.43; 10.18)		46	3.13 (2.11; 4.65)		
Single vessel disease	532	*Basal category*		339	*Basal category*		
Two vessel disease	402	1.30 (0.86; 1.96)	0.223	431	1.53 (1.13; 2.06)	0.006	0.371
Three vessel disease	359	2.10 (1.43; 3.09)	<0.001	594	2.24 (1.70; 2.95)	<0.001	0.508
Left main artery disease	11	4.12 (1.28; 13.28)	0.018	8	5.85 (2.50; 13.67)	<0.001	0.564
**IRA**							
Left main	18	4.52 (1.77; 11.57)	0.002	28	5.26 (3.26; 8.48)	<0.001	0.995
LAD incl. its branches	490	1.77 (1.17; 2.66)	0.006	556	1.10 (0.86; 1.41)	0.431	0.093
LCX incl. its branches	269	1.36 (0.83; 2.24)	0.222	221	1.09 (0.80; 1.48)	0.608	0.546
RCA incl. its branches	444	*Basal category*		401	*Basal category*		
Bypass graft	12	0.05 (0.00; 545.31)	0.524	20	0.80 (0.30; 2.17)	0.662	-
IRA not known	135	2.69 (1.63; 4.43)	<0.001	253	2.37 (1.83; 3.05)	<0.001	0.815

*N* number of patients in given category.

*HR* hazard ratio based on Cox proportional hazards model supplemented by its statistical significance.

Influence of age category of patients on HR value within these age categories is analyzed using interaction term in Cox proportional hazards model.

## Discussion

Though the age used for the definition of older or elderly patients varies among trials from 55 to 80 years [9-11] standard WHO definition of 65 years was applied for this study.

All patients were treated at academic tertiary hospitals, which provided 24/7 catheterizations. All reperfusion procedures, if indicated, were performed nearly solely by PCI; none of the patients, in either group, received thrombolysis. These conditions are unique in such a large group of consecutive, unselected patients having a diagnosis of acute myocardial infarction.

There is very limited data dealing with all types of AMI. However, in comparison with previously published data, the mortality in our cohort seems to be very low, especially in the older group. One of the reasons might be the exceptionally high catheterization (94.0%) and revascularization rate using PCI (79.9% of all study subjects). Despite the additional, known, risk factors and a worse expected prognosis in the older patients [12], the rate of diagnostic coronary angiography and PCI was found to be significantly lower in this high-risk population (92.4% vs. 95.9%, p < 0.001 and 74.7% vs. 85.6%, p < 0.001 respectively). One of the potential explanations for the lower PCI rate as well as the worse primary angiographic results in older patients might be the more complex and unfavorable anatomy. Unique data were collected from long-term survival analysis showing similar risk factors influencing the prognosis in both groups of patients with some exceptions. We clearly demonstrated age as a strong discriminating factor across the entire population of AMI patients. Though, for the most part not statistically significant, it seems to be clear that initial signs of heart failure (Killip II-IV), presence of diabetes mellitus and previous MI, final TIMI flow and the IRA are significant negative predictors but do not play as important a role in the older group as they do in younger patients. On the contrary, PCI in the older patients seems to be even more important than in younger patients (Table 3).

A comparison with any previously published data is rather difficult because of significantly lower catheterization and revascularization rate in previously published consecutive patient groups and a lack of analyzed cohorts of unselected consecutive patients with AMI. Mehta et al. [13] evaluated in-hospital mortality in STEMI patients (age ≥ 70 y) treated with thrombolysis and described higher mortality rates compared to our findings (14.4% in PCI-treated patients vs. 17.6% in patients treated with thrombolysis). Ishihara et al. [14] recently described the outcome of a large cohort of patients with AMI divided according to the age (< 70 years or ≥ 70 years). Despite the use of a favorable methodology, i.e. involving only patients undergoing catheterization within 24 hours from admission, the in-hospital mortality rate was substantially higher in both age groups comparing to our cohort (11.7% in the patients ≥ 70 years and 5.0% in patients < 70 years). Since age is a factor that cannot be changed, we have to focus on improvement of other modalities that can be influenced, such as shortening of the times between symptom onset and primary PCI [15]. Our findings strongly support the use of PCI in all patients (HR 0.60, p = 0.008 in younger patients and HR 0.46; p < 0.001 in older patients), which was also demonstrated by Nicolau et al. [16] in the ≥ 70 years population in adjusted models (HR 0.64, p = 0.001 older patients vs. HR 0.74, p = 0.073 younger patients).

### Study limitations

The study authors recognize the following limitations of the project. The registry is retrospective; however the cohort included unselected, consecutive patients with a diagnosis of AMI from multiple centers. The angiographic data were not assessed by an independent lab or in a blinded manner, although, angiographic findings were attained by experienced operators licensed in interventional cardiology. Only a limited number of coronary artery disease risk factors were followed and because of the comparison of two cohorts only univariate analysis was used to assess predictors of long-term survival.

## Conclusion

In a consecutive AMI population, the older group (> 65 years) was associated with a less pronounced impact of risk factors on long-term outcome. To ascertain the coronary anatomy by coronary angiography and proceed to PCI if suitable regardless of age is crucial in all patients, though the primary success rate of PCI in the older age is lower. Age, when viewed as a risk factor, was a dominant discriminating factor in all patients.

## Competing interests

The authors declare that they have no competing interests.

## Authors' contributions

All authors participated in data collection and processing, all authors read and approved the final manuscript.

## Pre-publication history

The pre-publication history for this paper can be accessed here:

http://www.biomedcentral.com/1471-2261/12/31/prepub
